# Chinese diabetes datasets for data-driven machine learning

**DOI:** 10.1038/s41597-023-01940-7

**Published:** 2023-01-19

**Authors:** Qinpei Zhao, Jinhao Zhu, Xuan Shen, Chuwen Lin, Yinjia Zhang, Yuxiang Liang, Baige Cao, Jiangfeng Li, Xiang Liu, Weixiong Rao, Congrong Wang

**Affiliations:** 1 School of Software Engineering, Tongji University, Shanghai, China; 2 AIway Oy, Helsinki, Finland; 3 Department of Endocrinology & Metabolism, Shanghai Fourth People’s Hospital, School of Medicine, Tongji University, Shanghai, China; 4 Department of Computer Science, School of Science, Aalto University, Helsinki, Finland; 5 Zhejiang Yugu Medical Technology Ltd, Zhejiang, China

**Keywords:** Public health, Machine learning, Metabolomics, Data mining

## Abstract

Data of the diabetes mellitus patients is essential in the study of diabetes management, especially when employing the data-driven machine learning methods into the management. To promote and facilitate the research in diabetes management, we have developed the *ShanghaiT1DM* and *ShanghaiT2DM* Datasets and made them publicly available for research purposes. This paper describes the datasets, which was acquired on Type 1 (n = 12) and Type 2 (n = 100) diabetic patients in Shanghai, China. The acquisition has been made in real-life conditions. The datasets contain the clinical characteristics, laboratory measurements and medications of the patients. Moreover, the continuous glucose monitoring readings with 3 to 14 days as a period together with the daily dietary information are also provided. The datasets can contribute to the development of data-driven algorithms/models and diabetes monitoring/managing technologies.

## Abstract (summary)

**Table:** 

Measurement(s)	blood glucose
Technology Type(s)	Continuous Glucose Monitoring System
Sample Characteristic - Organism	Homo sapiens

## Background & Summary

Diabetes is a chronic disease that could lead to cardiovascular disease, neuropathy, retinopathy, kidney failure and even mortality. Rapid socioeconomic changes and unhealthy lifestyle habits have led to the increasing prevalence of diabetes worldwide. Type 1 diabetes mellitus (T1DM) and Type 2 diabetes mellitus (T2DM) are the two main types of diabetes. T1DM is a chronic autoimmune disease resulting from destruction or damaging of the pancreatic beta cells^1^. T2DM is caused by insulin resistance and relative insulin deficiency^2^. T1DM accounts for only 5–10% of all diabetes worldwide, but varies geographically with the annual incidence of adult-onset T1DM about 1 per 100,000 in China^3^, while T2DM is the most common subtype of diabetes, accounting for over 90% of all the diabetes worldwide and in China^3,4^. It is shown that good blood glucose (BG) control significantly reduces the development or progression of chronic complications in T1DM and T2DM^5–7^. Thus, BG measurement plays a key part in diabetes care, which allows patients to adjust their food intake, physical activity and medications with the help of physicians (clinicians)^8^. Self-monitoring of blood glucose (SMBG) is a measurement that uses blood to collect blood glucose information at many time points^9^. Recently, a continuous glucose monitoring (CGM) technology is used to continuously monitor the BG levels in more or less real time^10,11^.

The use of CGM technology makes it possible to obtain a large amount of continuous BG data. However, there were relatively few publicly available BG datasets, as the data may have ethical restrictions and privacy concerns. There have been many studies^12,13^ on the BG prediction using different datasets. A rigorous literature review^12^ was conducted to develop a compact guide regarding machine learning methods on BG prediction in T1DM. The review included 55 papers from 2000 to 2018 and showed their subject, type of input, data source, input pre-processing methods, machine learning algorithms, prediction horizon and performance metrics. A systematical review^13^ on the literature from 2014 to 2020 was performed to study the data-based algorithms and models using real data for BG and hypoglycaemia prediction in T1/T2DM. The existing datasets in T1/T2DM for the BG prediction have been listed in the review. However, the T2DM datasets are much less studied than the T1DM datasets, e.g., 6 of 63 publications included T2DM in the review^13^. For real data, the data size was relatively small. In the review^13^, 27 papers (42.9%) present small samples (n < 10), 19 papers (30.2%) with small-medium samples (n = 11–50) and 17 papers (27%) with relatively large samples (n > 50). In another review for T1DM^12^, 51.7% were with small samples, 29.3% with small-medium samples, 17.2% with simulated data and 1.7% with samples over 50 patients. Another limitation pointed out by the reviews was the low free access data availability. Most data are credentialed or not accessible due to ethical restrictions and data privacy. We summarized recently studied and popular T1DM and T2DM datasets in Table 1.

**Table 1:** A summary on existing diabetes data in the literature.

Datasets	Type	Study period (days)	No.of patients	Data Availability	CGM /CBG	Food	Exercise	Insulin Use	Published Year
UVA/Padova^14,16–18,41,42^	T1DM	customized	30	Open^43^	✓/✓	✓	×	✓	2018
OhioT1DM^18,19,21,22,44^	T1DM	56	12	Credentialed^45^	✓/✓	✓	✓	✓	2020
D1NAMO^23^	T1DM	4	9	Credentialed^46^	✓/×	✓	✓	✓	2018
ABC4D^18,24^	T1DM	180	10	not accessible	✓/×	✓	×	✓	2020
Weinstock^25^	T1DM	12	201	not accessible	✓/×	×	×	✓	2016
KDD18^26^	T1DM	1095	40	Open^47^	✓/×	×	×	×	2018
Yang^29^	T1/T2DM	1–7	49/51	not accessible	✓/×	N/A	N/A	N/A	2018
Maryland^27^	T2DM	365	N/A	not accessible	×/✓	×	×	✓	2015
Maastricht study^28,30^	T2DM	2	851	Credentialed^48^	✓/✓	×	✓	×	2021

CBG, capillary blood glucose; CGM, continuous glucose monitoring; N/A, not available; T1DM, Type 1 diabetes mellitus; T2DM, Type 2 diabetes mellitus.

In T1DM, both real and simulated patient data in silico were well studied. Simulators can conveniently provide and customize detailed data of virtual diabetic patients from their dietary and treatment strategies. UVA/Padova T1DM simulator^14^ was widely employed, which was approved by Food and Drug Administration (FDA) and provided 30 different virtual patients freely. Virtual diabetes simulators were studied in tasks such as glycemic events identification, BG control^15^ and predictions^14,16–18^. The simulators were able to generate as many BG instances as possible for each patient^14^.

As a public dataset, *OhioT1DM*^18–22^ was a comprehensive dataset of real T1DM patients in the United States, which was publicly released by Ohio University and contained data of 12 real patients. Compared to the *OhioT1DM*, *D1NAMO*^23^ dataset focused on diabetes management. This dataset was composed of 20 real healthy people and nine real T1DM patients with additional patient information such as BG measurements, food pictures, breathing signals and accelerometer outputs. A clinical data^18,24^ including 10 T1DM adults from the *ABC4D* project using CGM sensors was used in a deep learning framework for accurate glucose forecasting. *Weinstock*^25^ collected diabetes-related data from adult type 1 diabetes (> = 60 years of age, diabetes duration > = 20 years). This dataset consisted of 14 days’ CGM data, information of insulin, other medications and patient demographics from 201 patients. This dataset was proposed to analyze the risk factors that can cause severe hypoglycemia in old patients. Fox *et al*.^26^ collected CGM records from 40 T1DM patients over three years (data size > = 1900 days of BG measurements, > = 550k distinct glucose measurements) and developed a deep multi-output forecasting algorithm.

T2DM datasets were less common than T1DM datasets^27,28^. A CGM data from both the T1DM and T2DM patients were employed to predict future BG levels for preventing hyperglycemia or hypoglycemia^29^, which was collected over a period ranging from 1.3 to 7 days. The *Maryland* data^27^ contained 56,000 SMBG data points collected in a 1-year prospective study. In this study, patients were treated with a variety of medications, including oral antihyperglycemic agents and insulin. The *Maastricht Study*^28,30^, an observational, prospective, population-based cohort study, focused on the aetiology, pathophysiology, complications and comorbidities of T2DM, and was characterized by an extensive phenotyping approach.

The existing diabetes data are used not only in BG prediction^31^, but also in other diabetes-related fields, such as the generation of BG control strategies^15^ and the study of the influence of external factors on blood glucose level. However, the limitations of many diabetes datasets in terms of the number of patients, the racial regions where they are collected, and the types of diabetes mellitus have led to the restrictions in diabetes-related research.

It is known that dietary intake, exercise and medication are the main factors affecting the BG level^32,33^. The collection on these external information is therefore essential in the datasets, which is a tedious task. More specifically, eating habits are quite influenced by ethnic groups and regions, e.g., the Chinese dietary habits are very complicated^34^. Therefore, two datasets from T1DM and T2DM patients in Shanghai, China with dietary information, clinical characteristics, laboratory measurements and medications of the patients were constructed. To the best of our knowledge, these are the first publicly available datasets to include rich information for people with T1DM and T2DM in China. The datasets could contribute to the research in data-driven machine learning.

## Methods

### Study population

A registry study on Diabetes Data Registry and Individualized Lifestyle Intervention (DiaDRIL) was initiated in Shanghai East Hospital and Shanghai Fourth People’s Hospital affiliated to Tongji University since 2019. The aims of this project were to provide evidence for personalized lifestyle recommendations and optimize glycemic control.

In this study, the patients were recruited from DiaDRIL in Shanghai East Hospital (September 2019 to March 2021) and Shanghai Fourth Peopleś Hospital (June 2021 to November 2021), respectively. The inclusion criteria were as follows: patients with diagnosed diabetes according to the 1999 World Health Organization (WHO) criteria; more than 18 years of age, willing to sign the informed consent form and with CGM recording for at least 3 days. Patients were excluded if they reported alcohol or drug abuse, were unable to comply with the study, or were not suitable to attend this study judged by the investigators. Data was anonymous to protect the sensitive information of the patients.

### Clinical and laboratory measurements

A standard questionnaire was conducted by trained research staff to obtain demographic information. Information on diagnosis and treatment of diabetes, duration of diabetes, laboratory measurements, comorbidities and pharmacologic treatments were collected from medical records. Each patient underwent a physical examination including measurement of height and weight. Body mass index (BMI) was calculated as weight divided by height squared (kg/*m*^2^). Each patient wore a flash glucose monitoring device (FreeStyle Libre H, Abbott Diabetes Care, Witney, UK) to measure interstitial glucose levels continuously for up to 14 days. CGM glucose data were automatically stored on the sensor every 15 minutes. The data can be obtained by scanning the glucose sensor with the reader and uploaded using the device software. Available laboratory measurements (≤6 months before or after CGM) including glucose metabolism, lipid profile and renal function were obtained from medical records. Any dietary intake including the exact time at consumption and weighed food record was reported by the patients. Hypoglycemic medications during CGM were also recorded.

This study was approved by the Ethics Committee of Shanghai Fourth People’s Hospital and Shanghai East Hospital affiliated to Tongji University in accordance with the Declaration of Helsinki. The informed consent was obtained from all the patients.

### CGM parameters

Time in range (TIR), one of the critical CGM-derived metrics, reflects the glucose variability and evaluates the quality of glycemic control^35^. It is associated with microvascular complications and macrovascular outcomes of diabetes. TIR is defined as the percentage of time spent in the target glucose range of 70–180 mg/dL. Time below range (TBR) and time above range (TAR) are the percentage of time when blood glucose is below 70 mg/dL and above 180 mg/dL, respectively. For most patients with T1DM or T2DM, the recommended CGM targets by the Advanced Technologies & Treatments for Diabetes (ATTD) consensus were ≥70% for TIR, ≤25% for TAR and ≤4% for TBR^36^.

### Analysis for CGM data

A clinical important task in diabetes management is the prevention of hypo/hyperglycemic events^37^. The algorithms to prevent the hpyo/hyperglycemic events can be obtained by generating hpyo/hyperalerts on the basis of ahead-of-time prediction of glucose concentration by using past CGM data and suitable time-series models.

Auto-correlation^38^ represents the degree of similarity between a given time series and a lagged version of itself over successive time intervals. It can help to uncover hidden patterns in data. Additionally, analyzing the autocorrelation function (ACF) and partial autocorrelation function (PACF) in conjunction is necessary for selecting the appropriate time-series models, e.g., ARIMA^39^.

$${\rho }_{k}=\frac{E\left[\left({x}_{t}-\mu \right)\left({x}_{t-k}-\mu \right)\right]}{{\sigma }^{2}}$$

where *x*_*t*_ is the observation at time *t*, *k* is lag, *E* is the expected value operator, *μ* is the mean and *σ*^2^ is the variance of the time series. *ρ*_*k*_ can show the correlation between two observations with a lag *k* in the time series.

## Data Records

The datasets *ShanghaiT1DM* and *ShanghaiT2DM* comprise two folders named “Shanghai_T1DM” and “Shanghai_T2DM” and two summary sheets named “Shanghai_T1DM_Summary” and “Shanghai_T2DM_Summary”. The datasets can be downloaded through *Figshare* repository^40^.

The “Shanghai_T1DM” folder and “Shanghai_T2DM” folder contain 3 to 14 days of CGM data corresponding to 12 patients with T1DM and 100 patients with T2DM, respectively. Of note, for one patient, there might be multiple periods of CGM recordings due to different visits to the hospital, which were stored in different excel tables. In fact, collecting data from different periods in one patient can reflect the changes of diabetes status during the follow-up. The excel table is named by the patient ID, period number and the start date of the CGM recording. Thus, for 12 patients with T1DM, there are 8 patients with 1 period of the CGM recording and 2 patients with 3 periods, totally equal to 16 excel tables in the “Shanghai_T1DM” folder. As for 100 patients with T2DM, there are 94 patients with 1 period of CGM recording, 6 patients with 2 periods, and 1 patient with 3 periods, amounting to 109 excel tables in the “Shanghai_T2DM” folder. Overall, the excel tables include CGM BG values every 15 minutes, capillary blood glucose (CBG) values, blood ketone, self-reported dietary intake, insulin doses and non-insulin hypoglycemic agents. The blood ketone was measured when diabetic ketoacidosis was suspected with a considerably high glucose level. Insulin administration includes continuous subcutaneous insulin infusion using insulin pump, multiple daily injections with insulin pen, and insulin that were given intravenously in case of an extremely high BG level.

Each excel table in the “Shanghai_T1DM” folder and “Shanghai_T2DM” folder contains the following data fields: <Date> Recording time of the CGM data. <CGM> CGM data recorded every 15 minutes. <CBG> CBG level measured by the glucose meter. <Blood ketone> Plasma-hydroxybutyrate measured with ketone test strips (Abbott Laboratories, Abbott Park, Illinois, USA). <Dietary intake> Self-reported time and weighed food intake <Insulin dose-s.c.> Subcutaneous insulin injection with insulin pen. <Insulin dose-i.v.> Dose of intravenous insulin infusion. <Non-insulin hypoglycemic agents> Hypoglycemic agents other than insulin. <CSII-bolus insulin> Dose of insulin delivered before a meal through insulin pump. <CSII-basal insulin> The rate (iu/per hour) at which basal insulin was continuously infused through insulin pump.

The summary sheets summarize the clinical characteristics, laboratory measurements and medications of the patients included in this study, with each row corresponding to one excel table in “Shanghai_T1DM” and “Shanghai_T2DM” folders. Clinical characteristics include patient ID, gender, age, height, weight, BMI, smoking and drinking history, type of diabetes, duration of diabetes, diabetic complications, comorbidities as well as occurrence of hypoglycemia. Laboratory measurements contain fasting and 2-hour postprandial plasma glucose/C-peptide/insulin, hemoglobin A1c (HbA1c), glycated albumin, total cholesterol, triglyceride, high-density lipoprotein cholesterol, low-density lipoprotein cholesterol, creatinine, estimated glomerular filtration rate, uric acid and blood urea nitrogen. Both hypoglycemic agents and medications given for other diseases before the CGM reading were also recorded.

## Technical Validation

### The characteristics of the Chinese diabetes datasets

The detailed characteristics of the patients in the *ShanghaiT1DM* and *ShanghaiT2DM* datasets were summarized in Table 2. The age of the *ShanghaiT1DM* group and the *ShanghaiT2DM* group was 57.8 ± 11.1 and 60.2 ± 13.7 years, respectively. There was no statistically significant difference in age between the *ShanghaiT1DM* group and *ShanghaiT2DM* group. This is because most of the patients (10/12) in the *ShanghaiT1DM* group belonged to a subtype of T1DM called “latent autoimmune diabetes in adults”, which is characterized by slow autoimmune *β*-cell destruction and an older mean age at onset of diabetes^1^. Women accounted for 58.3% of the *ShanghaiT1DM* group and 44% of the *ShanghaiT2DM* group, respectively. Besides, data concerning fasting plasma glucose, 2-hour postprandial plasma glucose and HbA1c were comparable between the two groups. However, the *ShanghaiT2DM* group had higher BMI values than the *ShanghaiT1DM* group (p < 0.05).

**Table 2:** The characteristics of the T1DM and T2DM patients in the ShanghaiT1DM and ShanghaiT2DM.

Characteristics	ShanghaiT1DM (n = 12)	ShanghaiT2DM (n = 100)	p value
Age, years	57.83 ± 11.12	60.17 ± 13.71	0.571
Women, n (%)	7 (58.3%)	44 (44.0%)	0.346
BMI, kg/*m*^2^	20.95 [17.87–24.21]	23.69 [22.12–25.54]	0.017
Duration of diabetes, years	8.50 [2.25–16.75]	7.00 [1.00–14.75]	0.614
Fasting plasma glucose, mg/dL	184.08 [117.00–262.35]	158.40 [126.00–194.40]	0.410
2-hour postprandial plasma glucose, mg/dL	297.00 [248.76–348.84]	250.65 [196.16–317.88]	0.218
HbA1c, mmol/mol	71 [63–122]	69 [54–97]	0.223

Data are presented as mean ± SD, median [interquartile range], or number(percentage%). BMI, body mass index; HbA1c, hemoglobin A1c; T1DM, Type 1 diabetes mellitus; T2DM, Type 2 diabetes mellitus.

**Fig. 1:**
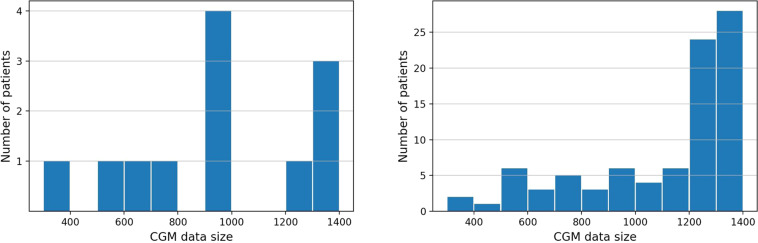
The number of recording files with different CGM data size in days (**a**) ShanghaiT1DM dataset (**b**) ShanghaiT2DM dataset.

To show the size of these two datasets more intuitively, we listed the patient’s type, the study period, sampling interval of CGM devices, number of patients, total number of recording files and total CGM measurements of the *ShanghaiT1DM* and *ShanghaiT2DM* in Table 3. For a given patient, he or she may have more than one recording period. In Fig. 1, we showed the number of recording files with different CGM data size in days in the *ShanghaiT1DM* and *ShanghaiT2DM*. The collected CGM data size varied from 3 days to 14 days.

**Table 3:** General characteristics of the datasets.

Datasets	Type of diabetes	Study period (days)	Monitoring interval (minutes)	No. of patients	No. of recording file	Total CGM measurements
ShanghaiT1DM	T1DM	4–14	15	12	16	15,695
ShanghaiT2DM	T2DM	3–14	15	100	109	112,475
SimulatorT1DM	T1DM	56	5	30	unlimited	482,610
OhioT1DM	T1DM	56	5	12	12	191,605

CGM, continuous glucose monitoring; T1DM, Type 1 diabetes mellitus; T2DM, Type 2 diabetes mellitus; No., number.

**Fig. 2:**
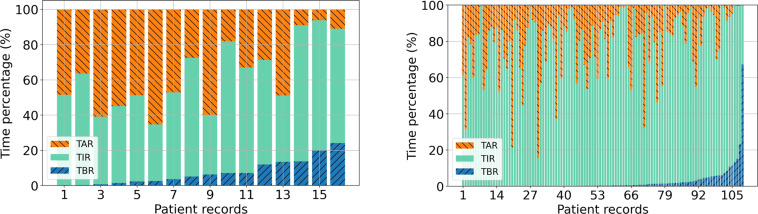
The average percentage of TBR (time below range), TIR (time in range) and TAR (time above range) for CGM in two datasets. (**a**) ShanghaiT1DM: TAR (37.8 ± 18.8%), TIR (54.7 ± 14.5%), TBR (7.5 ± 7.0%). (**b**) ShanghaiT2DM: TAR (20.0 ± 18.4%), TIR (77.7 ± 18.1%), TBR (2.4 ± 7.2%). Data are presented as mean ± SD.

We summarized the hypo/hyperglycemia events and calculated the auto-correlation coefficient on the BG values of the two datasets in time series. Hypoglycemia and hyperglycemia events are two potential risk factors for complications in diabetes. Hence, the time percentages of hypoglycemia (TBR) and hyperglycemia (TAR) events for each patient were calculated in Fig. 2. The horizontal axis represented each recording file of the patients with an order of TBR increasing, while the vertical axis represented the percentage of time (TAR, TIR and TBR) during the data collection period. The higher values of the TAR and TBR indicated that the patient’s condition was more serious. To give a clearer view of the TBR, TIR and TAR in the two datasets, we calculated the mean ± standard deviation of these values for the two datasets. For the *ShanghaiT1DM*, the mean ± standard deviation of the TIR were 54.7 ± 14.5% and 77.7 ± 18.1% for the *ShanghaiT2DM*. We noted that the average TIR was higher in T2DM patients than in T1DM patients (Fig. 2).

**Fig. 3:**
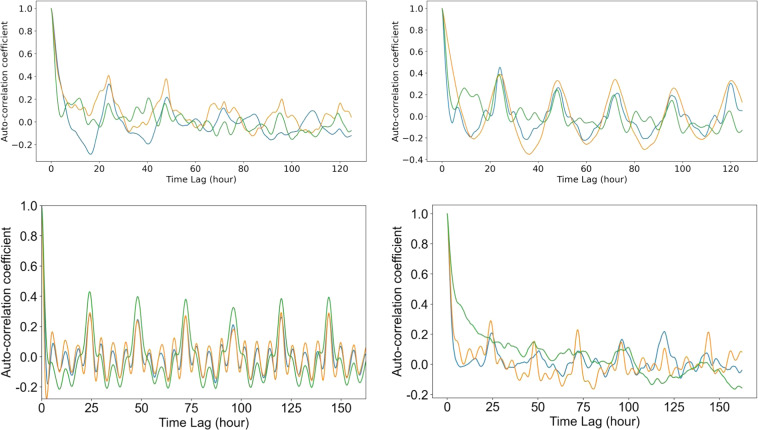
Auto-correlation coefficient of randomly picked three patients from the (**a**) ShanghaiT1DM, (**b**) ShanghaiT2DM, (**c**) SimulatorT1DM and (**d**) OhioT1DM.

Besides, as the collection on individual patient’s behavior information in each dataset was different, we randomly chose three patients from each dataset for the auto-correlation graph of the BG time series in Fig. 3. The auto-correlation coefficients identify seasonality and trend in time series data. It can be found that patients in *ShanghaiT2DM* (Fig. 3b) showed a more noticeable 24-hour periodic pattern than those in *ShanghaiT1DM* (Fig. 3a).

**Fig. 4:**
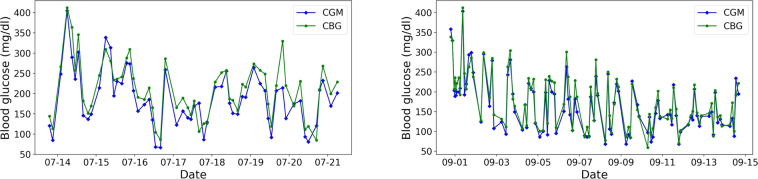
Randomly selected patients (**a**) 1008_0_20210713 and (**b**) 1003_0_20210831 in the ShanghaiT1DM for the distributions of glucose values of CGM readings and CBG. (CGM, continuous glucose monitoring; CBG, capillary blood glucose).

Since there might be discrepancy in BG levels by different blood glucose monitoring methods, we conducted a comparative analysis of the blood glucose measured by the CGM and CBG in Fig. 4, 5. The collection of the CBG was more sparse than that of the CGM, we only plotted the time stamps with both of the measurements. Two patients were randomly selected from each dataset. The results showed that the CBG values were usually greater than those of CGM readings.

**Fig. 5:**
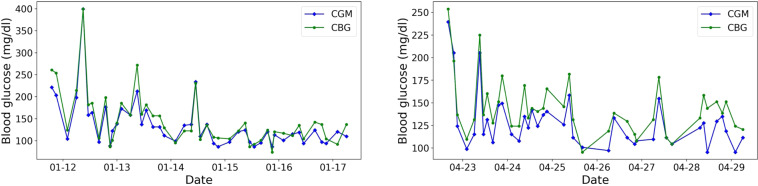
Randomly selected patients (**a**) 2010_0_20220111 and (**b**) 2022_0_20210419 in the ShanghaiT2DM for the distributions of glucose values of CGM readings and CBG. (CGM, continuous glucose monitoring; CBG, capillary blood glucose).

### Comparison to other datasets

**Fig. 6:**
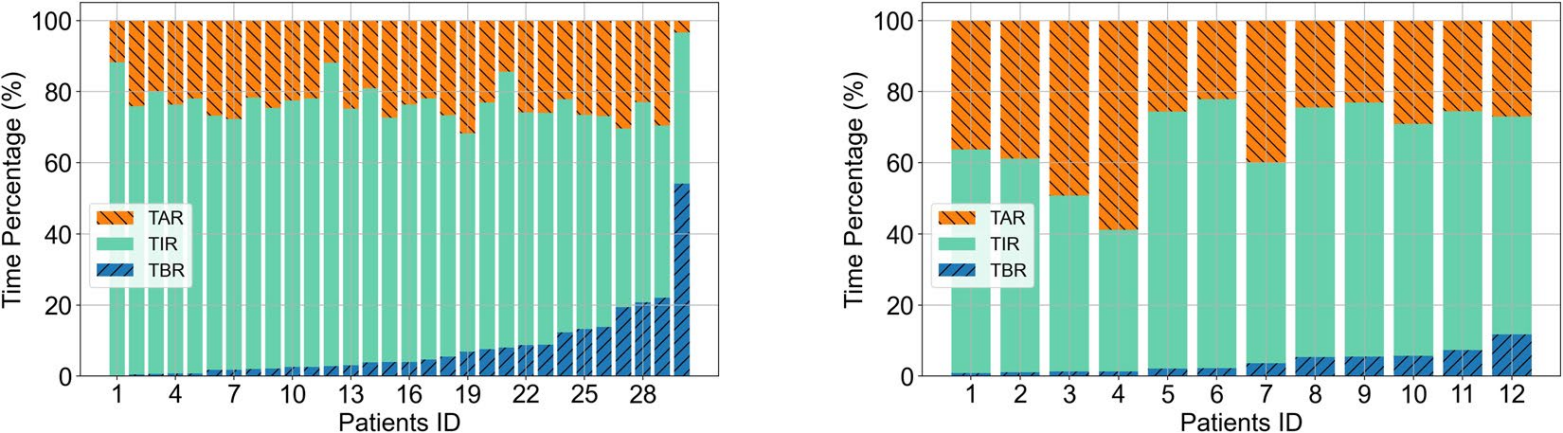
The average percentage of TBR (time below range), TIR (time in range) and TAR (time above range) for CGM (continuous glucose monitoring) in two datasets. (**a**) SimulatorT1DM: TAR (22.9 ± 5.9%), TIR (69.1 ± 10.2%),TBR (8.0 ± 10.5%). (**b**) OhioT1DM: TAR (33.4 ± 11.1%),TIR (62.6 ± 9.9%),TBR (4.0 ± 3.1%). Data are presented as mean ± SD.

There have been widely used datasets such as the *SimulatorT1DM* and the *OhioT1DM* (see Table 3). In order to show more specifically the difference between the newly constructed datasets and other existing data, the comparisons were performed in Table 3, figs. 3c,d & 6.

The auto-correlation coefficients of the *ShanghaiT1DM* (Fig. 3a) and *OhioT1DM* (Fig. 3d) indicated that the two real T1DM datasets shared similar trend and periodic pattern, which made it possible to combine the two datasets together in certain research. The *SimulatorT1DM* (Fig. 3c) had strong regularity as it was simulated.

Achieving higher TIR has been shown to reduce the percentages of time in the hypoglycemic and hyperglycemic range and complications of diabetes. In Fig. 6, we found that the patients in the *OhioT1DM* had lower mean TBR values compared to those in the *ShanghaiT1DM* (Fig. 2), which means that they have better control of hypoglycemia. In addition, patients in the *ShanghaiT2DM* (Fig. 2) had the highest mean TIR values, which suggests that people with T2D have better glycemic control overall than people with T1D. The virtual patients from the UVA/Padova (Fig. 6) had worse control of hypoglycemia, which may be due to the fact that the glycemic control strategy of the virtual patients was based on a fixed formula and therefore could not produce a timely response to the hypoglycemia. By comparing the *ShanghaiT1DM* and *OhioT1DM* (Fig. 6), we found that the standard deviations of TBR, TIR and TAR in the *ShanghaiT1DM* were higher than those in the *OhioT1DM*.

## Data Availability

The code for the analysis of the datasets and the generation of the figures and tables can be accessed in the Figshare repository^40^, which is a JUPYTER notebook named “data_analysis.ipynb”. The script can be executed with Python 3.6 and allows for reproducibility and code reuse.
